# Response to: “Proton-pump inhibitor use in combination with a CDK4/6 inhibitor: is formulation the issue?”

**DOI:** 10.1093/oncolo/oyag294

**Published:** 2026-07-31

**Authors:** Bo-Fang Chen, Chi-Cheng Huang

**Affiliations:** Division of Breast Surgery, Department of Surgery, Taipei Veterans General Hospital, Taipei, 112, Taiwan; Comprehensive Breast Health Center, Department of Surgery, Taipei Veterans General Hospital, Taipei, 112, Taiwan; School of Medicine, National Yang-Ming Chiao-Tung University, Taipei, 112, Taiwan; Division of Breast Surgery, Department of Surgery, Taipei Veterans General Hospital, Taipei, 112, Taiwan; Comprehensive Breast Health Center, Department of Surgery, Taipei Veterans General Hospital, Taipei, 112, Taiwan; Institute of epidemiology and preventive medicine, School of Public Health, College of Public Health, National Taiwan University, Taipei, 112, Taiwan

We thank Mr. Polwart for carefully reviewing our manuscript and for raising the important question about the palbociclib formulation. Mr. Polwart notes that palbociclib was reformulated from capsule to tablet in 2020 in some markets, unaffected by the intragastric environment, and that Schieber et al.’s data show co-administration with a proton pump inhibitor (PPI) does not significantly lower tablet bioavailability.^1^ We acknowledge that our original article did not explicitly specify which formulation was in use during the study period, and we appreciate this observation.^2^

The European Medicines Agency info for Ibrance shows that taking IBRANCE capsules with rabeprazole reduces palbociclib’s Cmax by 41% and AUCinf by 13% compared to IBRANCE alone. Under fasting conditions, AUCinf and Cmax drop by 62% and 80%, respectively.^3^ However, in tablet form, rabeprazole under fasting conditions does not alter absorption compared with IBRANCE alone^3^. This provides a strong rationale for transitioning to or testing the tablet formulation in clinical practice to mitigate interaction risks.

This resolution applies specifically to the formulation change of palbociclib. A study showed that PPI use doesn’t significantly affect ribociclib pharmacokinetics.^4^ According to Eli Lilly’s Monograph, abemaciclib can be taken with or without food, but it’s unclear if PPIs affect its absorption.^5^

Our study in Taiwan used the approved palbociclib capsule, available throughout data collection. The tablet formulation isn’t approved or marketed by the Taiwan FDA and wasn’t used in our patient population. The pharmacokinetic concerns about PPI reducing gastric pH and affecting palbociclib absorption apply to our cohort since the capsule’s solubility depends on pH, unlike the tablet. We agree this is a crucial distinction with significant implications for interpreting and applying our findings. Clinicians and pharmacists should be aware that the pharmacokinetic drug-drug interaction concern may differ with the tablet formulation. We agree that this nuance could be lost when our findings are simplified into drug interaction references without enough context.

We agree with Dr. Polwart that reading literature uncritically risks creating a generalized impression of a class-wide interaction between CDK4/6 inhibitors and PPIs across all formulations and markets. Takahashi et al.’s multicenter study with 78 patients on palbociclib, including 58 capsules and 20 tablets, showed no adverse interaction, conflicting with our research.^6^ However, Takahashi’s study defined “no concomitant use of PPIs” as PPI coverage for less than half of the CDK4/6i treatment, and “concomitant use” as at least half. These design differences may cause misclassification bias and affect comparability.

Regarding residual confounding from comorbidity scores, we share Mr. Polwart’s caution. As noted in our article, requiring a peptic ulcer diagnosis for PPI reimbursement in Taiwan’s National Health Insurance creates baseline group differences. We attempted to address this through propensity score adjustment but acknowledge residual confounding may exist, as higher comorbidity in PPI users could worsen outcomes. We state this limitation and do not claim causality.

We encourage future authors, guideline developers, and database curators to record drug formulation as a mandatory covariate in pharmacokinetic drug-drug interaction studies and to stratify conclusions accordingly. Currently, our findings are most relevant to the use of the palbociclib capsule formulation. We thank Mr. Polwart again for prompting this important clarification.

## References

[1] Schieber T , Steele S, Collins S, et al Effect of concurrent proton pump inhibitors with palbociclib tablets for metastatic breast cancer. Clin Breast Cancer. 2023;23:658-663. 10.1016/j.clbc.2023.05.009 37296062

[2] Chen BF , Lai JI, Tsai YF, et al Real-world evaluation of CDK4/6 inhibitors in hormone receptor-positive metastatic breast cancer: prognostic effect of proton pump inhibitor use. Oncologist. 2025;30:oyaf268. 10.1093/oncolo/oyaf268 PMC12505142 40891875

[3] EM Agency. Annex I summary of product characteristics. Accessed July 7, 2026. https://www.ema.europa.eu/en/documents/product-information/ibrance-epar-product-information_en.pdf

[4] Samant TS , Dhuria S, Lu Y, et al Ribociclib bioavailability is not affected by gastric pH changes or food intake: in silico and clinical evaluations. Clin Pharmacol Ther. 2018;104:374-383. 10.1002/cpt.940 29134635 PMC6099197

[5] ELC Inc. Verzenio (abemaciclib): Product Monograph. Updated January 22, 2026. Accessed July 7, 2026. https://pi.lilly.com/ca/verzenio-ca-pm.pdf

[6] Takahashi K , Uozumi R, Mukohara T, et al Proton pump inhibitors and cyclin-dependent kinase 4/6 inhibitors in patients with breast cancer. Oncologist. 2024;29:e741-e749. 10.1093/oncolo/oyae015 38340010 PMC11144975

